# Knowledge and attitude of final - year medical students in Germany towards palliative care - an interinstitutional questionnaire-based study

**DOI:** 10.1186/1472-684X-10-19

**Published:** 2011-11-23

**Authors:** Martin Weber, Sven Schmiedel, Friedemann Nauck, Bernd Alt-Epping

**Affiliations:** 1 Interdisciplinary Palliative Care Unit, III. Department of Medicine, University Medical Center of the Johannes Gutenberg University of Mainz, Germany; 2 Institute of Medical Biostatistics, Epidemiology and Informatics (IMBEI) at the University Medical Center of the Johannes Gutenberg University of Mainz, Germany; 3 Department of Palliative Medicine, University Medical Center, Göttingen, Germany

**Keywords:** Palliative care, knowledge, confidence, undergraduate curriculum

## Abstract

**Background:**

To care for terminally ill and dying patients requires a thorough medical education, encompassing skills, knowledge, and attitudes in the field of palliative care. Undergraduate medical students in Germany will receive mandatory teaching in palliative care in the near future driven by recent changes in the Medical Licensure Act. Before new curricula can be implemented, the knowledge of medical students with respect to palliative care, their confidence to handle palliative care situations correctly, their therapeutic attitude, and their subjective assessment about previous teaching practices have to be better understood.

**Method:**

We designed a composite, three-step questionnaire (self estimation of confidence, knowledge questions, and opinion on the actual and future medical curriculum) conducted online of final - year medical students at two universities in Germany.

**Results:**

From a total of 318 enrolled students, 101 responded and described limited confidence in dealing with specific palliative care issues, except for pain therapy. With regard to questions examining their knowledge base in palliative care, only one third of the students (33%) answered more than half of the questions correctly. Only a small percentage of students stated they had gained sufficient knowledge and experience in palliative care during their studies, and the vast majority supported the introduction of palliative care as a mandatory part of the undergraduate curriculum.

**Conclusion:**

This study identifies medical students' limited confidence and knowledge base in palliative care in 2 German universities, and underlines the importance of providing a mandatory palliative care curriculum.

## Background

The necessity of improving palliative care for patients with advanced and incurable diseases has been increasingly acknowledged over the last two decades in Germany, and is now sustained by broad societal consensus [1]. Additionally, the German Medical Association has commented on palliative care in several national assemblies and has strongly requested improvements in several areas, including medical education [2]. Until recently, palliative care was not a mandatory part of the undergraduate curriculum, and it remained the responsibility of the medical schools to offer courses in palliative care. Only a few of the 35 medical schools in Germany included mandatory courses, whereas the vast majority did not provide comprehensive palliative care education [3,4].

However, as a consequence of changes in the Medical Licensure Act of 2009 (ÄApprO as of 03.07.2002, last amended in 31.07.2009, §27 and supplement 15 to §29 sect. 3 sentence 2), palliative care has become a mandatory part of the undergraduate medical curriculum [5,6]. This new legislation must be fully implemented by the spring of 2013.

Whereas the insufficient preparation of German medical students in the care of terminally ill and dying patients has been discussed and reflected on by a number of authors [7-9], data describing the students' actual knowledge, skills, and attitudes towards palliative care remain scarce [10,11]. The evaluation of a non-mandatory course showed an increase in knowledge of pain management and symptom control, as rated by self-assessment, of first-, third - and fifth-year medical students. However, improvement with respect to the accompaniment of dying patients, communicating bad news, the integration of spiritual aspects, as well as the overall results at the end of undergraduate training, were found to be poor [11].

We therefore performed a questionnaire-based study in order to evaluate the knowledge base in palliative care of final - year medical students, including their sense of confidence in dealing with palliative care issues, their attitudes towards specific end - of - life care situations, and their subjective assessment of their curriculum before palliative care was introduced as a mandatory part of undergraduate teaching.

The students were recruited from two universities which had no mandatory palliative care teaching in the curriculum, shortly before the implementation process started.

## Methods

### Development of the questionnaire

An interdisciplinary panel of experts in palliative care developed a three - step questionnaire for final - year students. This questionnaire is based on two methodological elements that have been used in previous studies:

• The first part inquired about the students' feelings of confidence towards ten relevant aspects in palliative care, assessed by self-estimation, and related to the above study of first -, second -, and fifth - year medical students at the Universities of Cologne and Bonn [11]. Whilst the original questionnaire was comprised of nine items, our modified version was comprised of ten items, as the "spiritual and psychological aspects" section was split into two separate items. The degree of "confidence" was differentiated in a semiquantitative manner on a four - step Likert scale (confident - rather confident - rather non confident - non confident).

• The second part gathered detailed information about the knowledge level of the final - year students, relying on the validated "Palliative Care Examination" tool developed by Weissman et al. [12,13]. Ten brief case reports were given for which 36 multiple choice questions (MCQs) were asked. The topics comprised pain therapy (50% of all MCQs), symptoms other than pain (17%), psychosocial aspects (17%), health policy or organizational issues (6%), prognostication (3%), and ethics (8%). The case reports and contents were then adapted to the medical conditions in Germany (e.g. drugs). As two case reports could not be transferred to local conditions (focusing on health policy and organization), the second part was abridged to eight case examples and 21 MCQs, leaving content percentages largely unchanged: pain, 57%, psychosocial aspects, 19%, symptoms other than pain, 19%, and ethics issues, 5%.

• In the third part, the students were asked about their experiences concerning the teaching of palliative care in the current curriculum, and whether they considered the mandatory implementation of palliative care into the curriculum a reasonable step.

This three - step questionnaire was pretested by ten medical students with respect to comprehensibility, acceptance, duration, and handling. Finally, the questionnaire was transformed into an online version.

The study was performed at the University of Mainz and the University Medical Center Göttingen. All students obtained written information in advance and a link to the online questionnaire. Non-responders received a maximum of three e-mails as reminders.

According to local ethics standards no formal ethical approval was requested.

### Statistical data processing

SAS V9.2 was used for the statistical analysis. Data was stored anonymously and underwent descriptive statistical analysis. Scores were calculated within categories of answers. In the first part of the questionnaire, these were: total score, somatic score (questions 1, 4 and 6), psychosocial score (questions 3, 7, 8, 9, and 10) and spiritual score (question 5). The following scores were formed in the second part of the questionnaire: pain knowledge (questions 1 - 12), psychosocial knowledge (questions 13, 14, 20, and 21), knowledge about ethics (question 15), and symptom control knowledge (questions 16 - 19). A two-sided t - test was used assuming different variances between groups.

## Results

Overall, 318 (Mainz 155, Göttingen 163) students were approached to participate in the study. Of these, 101 students (31%) answered the questionnaire (76 from Mainz, 49%; 25 from Göttingen, 15%).

Table 1 lists the answers to the questions from the first part of the questionnaire (self-assessment), overall and separately for the Universities of Mainz and Göttingen. Overall, only 5% to 10% of students declared a high level of confidence in dealing with palliative care issues. Only pain management was approached with confidence by more than 50% of students (69% in Mainz were "confident" and "rather confident", but only 24% in Göttingen). The answers to all the other questions showed a lack of self-confidence.

**Table 1:** Self assessment of confidence in different domains of palliative care, overall (n = 101) and separately for the universities of Mainz (n = 76) and Göttingen (n = 25)

question	university	confident n (%)	rather confident n (%)	rather non confident n (%)	non confident n (%)	n =
(1)Regarding assessment and examination of patients with cancer pain I feel...	overall Mainz Göttingen	2 (2) 1 (1) 1 (4)	34 (34) 28 (37) 6 (24)	57 (56) 43 (57) 14 (56)	8 (8) 4 (5) 4 (16)	101 76 25

(2) Regarding the basic principles and contents of palliative care I feel...	overall Mainz Göttingen	3 (3) 3 (4) 0	30 (30) 20 (26) 10 (40)	59 (58) 46 (61) 13 (52)	9 (9) 7 (9) 2 (8)	101 76 25

(3) Regarding the integration of psychological aspects in the treatment and accompaniment of severely ill and dying patients I feel...	overall Mainz Göttingen	5 (5) 4 (5) 1 (4)	30 (30) 24 (31) 7 (28)	52 (51) 42 (55) 10 (40)	14 (14) 7 (9) 7 (28)	101 76 25

(4) Regarding the medical treatment of cancer pain I feel...	overall Mainz Göttingen	8 (8) 8 (11) 0	49 (49) 44 (58) 6 (24)	29 (29) 20 (26) 9 (36)	14 (14) 4 (5) 10 (40)	101 76 25

(5) Regarding the integration of spiritual aspects in the treatment and accompaniment of severely ill and dying patients I feel...	overall Mainz Göttingen	5 (5) 5 (7) 0	17 (17) 13 (17) 4 (16)	45 (45) 38 (50) 8 (32)	33 (33) 20 (26) 13 (52)	101 76 25

(6) Regarding the treatment of symptoms, which might occur in advanced cancer, I feel...	overall Mainz Göttingen	4 (4) 3 (4) 1 (4)	41 (41) 32 (42) 9 (38)	45 (45) 34 (45) 11 (45)	10 (10) 7 (9) 3 (13)	100 76 24

(7) Regarding communicating with severely ill and dying patients I feel...	overall Mainz Göttingen	7 (7) 5 (7) 2 (8)	36 (36) 29 (38) 7 (28)	40 (40) 30 (40) 10 (40)	17 (17) 11 (15) 6 (24)	101 76 25

(8) In explaining to a patient the inability to cure her/his disease, I feel...	overall Mainz Göttingen	2 (2) 1 (1) 1 (4)	17 (17) 12 (16) 5 (20)	44 (44) 36 (48) 8 (32)	37 (37) 26 (35) 11 (44)	100 75 25

(9) In explaining to a patient the change from a tumor specific treatment (e.g. chemotherapy) to palliative care I feel...	overall Mainz Göttingen	1 (1) 1 (1) 0	19 (19) 14 (19) 5 (20)	60 (59) 45 (59) 15 (60)	21 (21) 16 (21) 5 (20)	101 76 25

(10) Regarding the treatment of and accompaniment of terminally ill and dying patients I feel...	overall Mainz Göttingen	0 0 0	20 (20) 17 (23) 3 (12)	51 (50) 39 (51) 12 (48)	30 (30) 20 (26) 10 (40)	101 76 25

**Figure 1:**
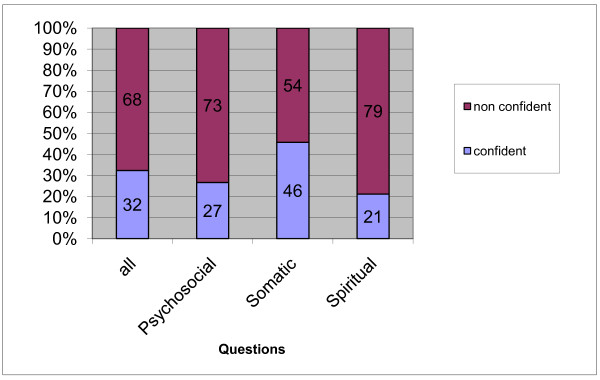
**Extrapolated self estimation (percentages) of confidence in relation to four topic scores for both universities (Mainz and Göttingen) combined**.

The questions were categorized as described in the Methods section. Figure 1 differentiates four topic scores and comprises the according sense of confidence into two categories ("confident" and "rather confident" versus "rather non confident" and "non confident") for both universities combined. Students were noticeably less confident about questions regarding spiritual and psychosocial matters.

The second part of the questionnaire was comprised of 21 questions exploring knowledge. One third of the responding students (33%) answered more than half of the questions (eleven or more) correctly (Table 2).

**Table 2:** Correct answers to 21 questions exploring knowledge in percent of responding students

	Mainz (%)	Göttingen (%)	Overall (%)	missing answers (abs)
Identify somatic pain	70	60	68	2

Move to strong opioid	62	24	53	2

NSAID for bone pain	62	12	49	2

Treatment of breakthrough pain	66	21	58	2

Conversion oral to IV morphine	51	36	47	2

Time to therapeutic for fentanyl patch	41	36	39	2

Equianalgetic dose of fentanyl	23	16	21	2

Opioid nausea resolves in < 7 days	65	52	62	2

Side-effects of opioids	29	20	27	3

Worsened pain is worsened cancer	19	24	20	3

Identify neuropathic pain	67	42	61	5

Tricyclics for AIDS pain	51	25	44	5

Family says: don't tell	76	75	76	6

Tell prognosis in cancer	49	46	48	6

Define physician assisted suicide and euthanasia	48	75	55	6

Treat dyspnoea	39	38	39	6

Diagnose hypercalcaemia	23	4	18	7

Treat death rattle	21	9	18	7

No IV hydration for dying patient	34	43	36	7

Pronounce death	58	57	57	7

Identify normal grief	54	61	56	6

Only 43% of the questions that explored the knowledge of facts were answered correctly (Table 3). At the University of Mainz, 49% of the final - year students answered the MCQ concerning pain therapy correctly, and only 31% at Göttingen. The only MCQ related to ethics was answered correctly by 72% in Göttingen, and in Mainz, by 45%. In respect to the different scenarios included in this question, 27% of the responding students (31% in Mainz, 17% in Göttingen) were of the opinion that termination of hydration in a dying patient is a mode of euthanasia. Furthermore, 37% of the students thought that the termination of artificial ventilation at the request of a competent patient is not allowed (45% in Mainz, 13% in Göttingen), and only 55% of all students could correctly identify the definition of euthanasia. The knowledge of how to treat symptoms other than pain was especially low (26%). Students that estimated themselves as "confident" scored better in the MCQ related to pain therapy compared to students who estimated themselves as "non confident", but their rate of correct answers were still rather poor (53% versus 34%; p < 0.05).

**Table 3:** Questions exploring knowledge (percentage of correct answers, SD = standard deviation)

	overall mean (SD)	Mainz mean (SD)	Göttingen mean (SD)	male n = 25	female n = 41	number of questions
Pain score	45% (21%)	49% (21%)	31% (14%)	48% (20%)	46% (21%)	12 questions

Psychosocial score	56% (26%)	56% (28%)	56% (23%)	50% (23%)	63% (26%)	4 question

Ethics score	51% (50%)	45% (50%)	72% (46%)	44% (51%)	61% (49%)	1 questions

Symptom score	26% (24%)	27% (25%)	22% (21%)	27% (24%)	26% (25%)	4 questions

Overall	43% (17%)	46% (18%)	36% (12%)	44% (17%)	46% (17%)	21 questions

Only a small proportion of students agreed to the statement that it was true or rather true that they had gained knowledge and experience in palliative care during their studies (overall 25%; Mainz 27%; Göttingen 21%), and the vast majority of students supported the introduction of palliative care as a mandatory part of the undergraduate curriculum (overall 94%; Mainz 94%; Göttingen 92%).

## Discussion

This questionnaire-based study provides further evidence of both medical students' limited confidence in dealing with palliative care issues, and in general, their limited knowledge in the field of palliative care at universities that do not provide a comprehensive mandatory curriculum. This study innovatively combines self-assessment, a knowledge-based examination, and the students' perspective on curricular developments at the advent of legislative changes in Germany that will include mandatory undergraduate palliative care teaching.

Medical students self-reflected on their own incapacities in all relevant fields of palliative care (first part of the questionnaire), and immediately provided a proof of concept with their low scores in the second part of the questionnaire, where knowledge on palliative care issues was required. Only the self-assessment on pain therapy was more positive than on other subjects. However, the scores achieved on the pain - related MCQs did not accordingly correspond with the students' assessments. Interestingly, pharmacology of pain therapy was an integral part of the curriculum at both study sites shortly before the final year began.

The degree of confidence with respect to psychosocial issues was shown to be extraordinarily low; 80% of the medical students stated that they were "rather non confident" or "non confident" with respect to the accompaniment of severely ill or dying patients, and 57% with respect to communication in the palliative care setting. Seventy - three percent were "rather non confident" or "non confident" regarding all questions addressing psychosocial issues.

The weighing of ethical problems also proved to be deficient (MCQ # 15); this also referred to the understanding of common and basic treatment modalities, such as refraining from IV volume substitution or terminating artificial ventilation, that have been commonly misunderstood as illegal euthanasia. It remains speculative whether the mandatory courses in medical ethics shortly before the final year begins contributed to a relatively better score at the Göttingen study site. In a previous study six years earlier which explored medical students' attitudes towards ethical decision making, the problem of misunderstanding the term euthanasia proved to be even more prominent (32% with respect to terminating IV hydration; 46% with respect to discontinuing artificial ventilation) [14]. This misunderstanding appears to be even more problematic in the light of current discussions about legalizing end-of-life practices like physician - assisted suicide or even euthanasia in Germany.

Spiritual care proved to be a major obstacle, as only a sixth of the study participants felt confident in this area.

A lack of confidence and knowledge in palliative care is not limited to German medical students. The "Palliative Care Examination", a test that served as a draft for the second part of our questionnaire, was performed on 32 first year interns in US hospitals (44% correct answers), and residents and attending doctors (58% correct answers). The questions concerning pain assessment and management were scored correctly in 46% and 57%, respectively [12]. In another study, 39% of medical students denied being well prepared to talk with patients about their anxious thoughts and death [15]. Fraser et al. questioned senior medical students of six medical schools concerning their education in palliative care, only 50% felt sufficiently prepared to treat common end - of - life symptoms or to communicate adequately in this setting (34%) [16].

The vast majority of our study participants (> 90%) would welcome the implementation of mandatory courses in palliative care. This finding is quite remarkable, as in June 2010, the *Medizinische Fakultätentag der Bundesrepublik Deutschland *declined to take a stand in favor of a mandatory implementation of palliative care into the undergraduate curriculum, as such a regulation would be superfluous; palliative care would be already covered by curricular legislation, and medical schools were already authorized to implement courses "on demand" [press release 4.6.2009, http://idw-online.de/pages/de/news318654; accessed 14.6.2010].

A recently published study of United States medical schools suggests a similar situation. A minority of US medical schools, from which information was obtained, required formal training in palliative care and evaluated students in their care of patients with advanced, incurable conditions. The majority chose to include palliative care topics within existing courses [17]. While the British General Medical Council has clearly recommended that all medical students should receive core teaching on relieving pain and distress, and in caring for the terminally ill, surveys have shown that the palliative care curriculum provided by British medical schools varies widely, ranging from 6 to 100 hours [18]. In a comparative study published in 2007, 85% of final - year British students, but only 25% of US students, reported having taken a course or clinical rotation in end-of-life care [19]. Whereas the optimal structure and content of formal palliative care teaching is still a matter of debate [20,21], several studies have described the positive effects of such mandatory teaching [22,23]. Due to the new legislation, undergraduate palliative care education in Germany will undergo significant changes during the next three years. This process provides a unique opportunity to evaluate the impact of a new mandatory curriculum for all German medical schools. Our study gives a snap-shot of the current situation and provides a convenient instrument which can be repeated during and after the implementation process.

### Limitations

The response rate of 31% (Mainz 49%, Göttingen 14%) was rather low, especially at the Göttingen study site, despite several efforts to improve recruitment. Therefore, scoring differences between the two study sites cannot easily lead back to differences between the two existing curricula, and results cannot be considered to be representative of all medical students in Germany. Considering the possibility that rather motivated students responded, it is possible that scores would have been even more disappointing if the overall response rate had been higher. Furthermore, a biased response behavior towards desired answers in the third part of the questionnaire cannot be excluded.

## Conclusions

Final year German medical students from 2 German universities were found to be insufficiently prepared to care for terminally ill and dying patients as self-assessed in their own level of confidence and via a knowledge - based questionnaire accordingly. Their strong vote in favor of a mandatory curriculum in palliative care gained recognition by an analogous legislation that must be completed by 2013.

## Competing interests

The authors declare that they have no competing interests.

## Authors' contributions

MW conceived of the study, participated in its design and coordination and drafted the manuscript. SS performed the statistical analysis and drafted the presentation of the results. FN participated in the design of the study and the critical revision of the manuscript. BAE participated in the design of the study, in data collection at the Göttingen study site, and in drafting the manuscript. All authors read and approved the final manuscript.

## Pre-publication history

The pre-publication history for this paper can be accessed here:

http://www.biomedcentral.com/1472-684X/10/19/prepub
